# Impact of First Aid on Treatment Outcomes for Non-Fatal Injuries in Rural Bangladesh: Findings from an Injury and Demographic Census

**DOI:** 10.3390/ijerph14070762

**Published:** 2017-07-12

**Authors:** Dewan Md Emdadul Hoque, Md Irteja Islam, Shumona Sharmin Salam, Qazi Sadeq-ur Rahman, Priyanka Agrawal, Aminur Rahman, Fazlur Rahman, Shams El-Arifeen, Adnan A. Hyder, Olakunle Alonge

**Affiliations:** 1 Maternal and Child Health Division, International Centre for Diarrheal Diseases Research, GPO Box 128, Dhaka 1000, Bangladesh; irteja.islam@icddrb.org (M.I.I.); shumona@icddrb.org (S.S.S.); qsrahman@icddrb.org (Q.S.-u.R.); shams@icddrb.org (S.E.-A.); 2 Johns Hopkins International Injury Research Unit, Department of International Health, Johns Hopkins Bloomberg School of Public Health, Baltimore, MD 21205, USA; pagrawa6@jhu.edu (P.A.); ahyder1@jhu.edu (A.A.H.); oalonge1@jhu.edu (O.A.); 3 Centre for Injury Prevention and Research, House #B-162, Road #23, New DOHS, Mohakhali, Dhaka 1206, Bangladesh; aminur@ciprb.org (A.R.); fazlur@ciprb.org (F.R.)

**Keywords:** non-fatal injury, first-aid treatment, medically trained providers, untrained medical providers, rural, Bangladesh

## Abstract

Non-fatal injuries have a significant impact on disability, productivity, and economic cost, and first-aid can play an important role in improving non-fatal injury outcomes. Data collected from a census conducted as part of a drowning prevention project in Bangladesh was used to quantify the impact of first-aid provided by trained and untrained providers on non-fatal injuries. The census covered approximately 1.2 million people from 7 sub-districts of Bangladesh. Around 10% individuals reported an injury event in the six-month recall period. The most common injuries were falls (39%) and cuts injuries (23.4%). Overall, 81.7% of those with non-fatal injuries received first aid from a provider of whom 79.9% were non-medically trained. Individuals who received first-aid from a medically trained provider had more severe injuries and were 1.28 times more likely to show improvement or recover compared to those who received first-aid from an untrained provider. In Bangladesh, first-aid for non-fatal injuries are primarily provided by untrained providers. Given the large number of untrained providers and the known benefits of first aid to overcome morbidities associated with non-fatal injuries, public health interventions should be designed and implemented to train and improve skills of untrained providers.

## 1. Introduction

Injuries are a relatively neglected health issue, [1,2,3,4] around 4.7 million people die annually as a result of intentional and unintentional injuries which together account for 8.5% of all deaths globally [5,6]. In 2010, an estimated 11% of the total cause of disability-adjusted life years (DALYs) was attributed to injuries with over 90% of the DALYs lost occurring in low- and middle-income countries (LMICs) [7,8]. Non-fatal injuries occur more often than fatal injuries and have a significant impact on disability, productivity, cost of treatment and rehabilitation [9,10,11]. It has been forecast that the magnitude of both non-fatal and fatal injuries will decline in high-income countries, but will continue to be a significant cause of death and disability in the developing world over the next 20 years [1,8]. In LMICs, injuries account for about one third of all outpatient hospital visits [7,12]. Despite its overall significance, the burden of injuries in these countries has not yet been fully understood due to lack of population-based data at a country level leading to inadequate preventive efforts, limited resources and ill-equipped healthcare systems to address the issue [1,3,13].

In Bangladesh, sparse data exist to quantify the burden of injuries at the community level. The 2003 Bangladesh Health and Injury Survey (BHIS) indicated that injuries were the greatest killer for children 1 to 18 years of age. According to the BHIS, over 30,000 Bangladeshi children died from injury in 2004, about three children per hour [14]. Drowning, road traffic incidents, falls and burns are among the most common causes of injury in Bangladesh [15,16].

Provision of first aid for injuries is a secondary preventive measures taken immediately after an injury event by trained clinicians and first responders, resulting in better outcomes for injured victims. The International Federation of Red Cross and Red Crescent Societies (IFRC) states that while first aid is by no means a substitute for emergency health services, it is a pivotal primary step for providing effective and rapid interventions to reduce serious injuries and increase the chances of survival [14]. To be most effective, first aid should be provided immediately after the event. For example, effective bystander cardiopulmonary resuscitation (CPR) provided immediately after cardiac arrest can double a person’s chance of survival as it helps maintain vital blood flow to the heart and brain [14]. Also, the immediate application of running cold water for 20 min, can stop the burn process and positively affect the outcome of burns [17,18]. Studies conducted in developed countries on non-fatal injuries have reported first aid to play a significant role in reducing mortality rates [19]. In developing countries, several studies have shown that first aid given by an untrained provider (e.g., caregiver, bystander) or a trained provider is increasingly essential to reduce mortality as well as severity of injuries [19,20,21,22]. Research on severe non-fatal injuries such as burns, blunt trauma and road traffic incidents in high-income settings has found significant reduction in mortality rates when first aid was applied [19,22,23]. Despite the large burden of injuries in LMICs and the importance of first aid in decreasing injury severity and increasing survival, there is a dearth of research in LMICs like Bangladesh around the subject [1,3]. Moreover, the few available studies in LMICs are hospital-based and suggest that a significant proportion of patients with non-fatal injury events did not receive first aid treatment from any health care facility [24]. Therefore, the objective of this study was to quantify the impact of first aid provided by trained and untrained providers on severe, non-fatal injuries in rural Bangladesh using population-based data collected from a baseline census conducted in 2013 as part of a drowning prevention study.

## 2. Materials and Methods

### 2.1. Study Design, Area and Population

This paper is based on data collected as part of a large-scale implementation study, “Saving of children’s Lives from Drowning” (SOLID) project [25,26]. A cross-sectional baseline census was conducted over a period of six months (June to November 2013) prior to implementing a package of drowning prevention interventions in seven rural sub-districts of Bangladesh. The baseline census covered approximately 1.16 million people (based on the 2011 Bangladesh National Census) across 51 unions from Matlab North, Matlab South, Daudkandi, Chandpur Sadar, Manohardi, Raiganj and Sherpur Sadar. Unions are the lowest administrative unit of local government in Bangladesh [27].

### 2.2. Questionnaire and Data Collection

The baseline census collected information on socio-demographic details, injury events, first aid practices and health care seeking behaviors for all injury events and outcomes on all populations in selected sub-districts. Data was collected using a structured, pre-tested questionnaire and consisted of seven modules. Specific questions related to first aid practices and health care seeking behaviors were considered in the injury morbidity (module V) and injury mortality (module VI) modules. All non-fatal injury related information was collected over a six-month recall period; however, deaths were collected over a one-year recall period. Face-to-face interviews were conducted with the household head or any household member 18 years and older to collect all required information. All the tools were written in English and translated to Bangla and written informed consent was obtained from all respondents [27]. The survey was implemented such that the “don’t know” where later confirmed as a “no” based on the follow-up questions asked by the interviewer. The instruction that was given to the interviewer was that they needed to clarify if any treatment was obtained by the injured person, and all those who responded no or don’t know were asked some follow-up questions such as if the injured was taken to a hospital, or healthcare provider, or if any interventions was administered to help ascertain that they in fact did not receive any treatment.

Non-fatal injury was defined as “any household member who sought treatment or lost at least one working day or could not go to the school for at least one day due to any of injury events”. First-aid treatment was defined as “any household member who received emergency care (from medically trained or untrained provider) immediately after the injury and prior to full medical treatment, if treatment was sought”. Health care seeking behavior was defined as “any household member who sought first aid treatment or any type of surgical or medical intervention either from trained health care provider or untrained provider”. Registered medical doctors and nurses were considered as trained providers whereas any other person, such as friends, peers, village doctors, or relatives were considered as untrained providers. Information on the treatment outcomes after first aid was also obtained. For each participant reporting an injury event, an injury severity score was calculated based on principal component analysis on eight variables—anatomic and physiologic profiles of an injury, post injury immobility, post-injury hospitalization, surgical treatment, post-injury disability, number of days an individual required assistance, and the number of days lost at work or school. The injury severity scores were categorized into severity tertiles that correspond with low, medium and high severity categories [27]. In addition, treatment outcomes were described for all non-fatal hospitalized injuries, and these were categorized into no improvement, recovering or fully functional and anatomic recovery. ‘Fully recovered’ is defined as anybody who has reported to have regained full physiological and anatomical functionality of the part of the body that was injured. If the physiological and anatomical functionality is better than when the injury took place, but not at the level experienced prior to the injury, it was classified as ‘improving’. If the physiological and anatomically functionality remains at the same level as it was during the injury, then it was categorized as ‘no improvement’. This was a self-reported information obtained based on the perception of the injured individuals regarding their state irrespective of their injury severity or whether they had received first aid or not.

### 2.3. Statistical Method and Analyses

Counts and frequencies of non-fatal injuries were calculated and categorized under each injury severity categories: low, medium and high severity. The counts and frequencies under each injury severity categories were further described by whether the individuals received first aid or not. Counts and frequencies were calculated for those that received first aid, and these were described by age, sex, external causes of injury, occupation, educational attainment, geographical area and type of provider (medically trained compared to untrained).

For all injuries categorized under the high severity category and for which first aid was provided, the association between treatment outcomes and types of service provider were assessed using multivariate logistic regression models, and adjusted for key covariates including the external causes, educational level, occupation, wealth quintile, age, sex and geographic area of each household member. All estimations were reported as odds ratios (OR), with their respective 95% confidence intervals (CI). Variable construction and estimations were done with statistical software STATA V.13 (Stata Corp., College Station, TX, USA).

### 2.4. Ethical Approval

Ethical approval for the study was obtained from the Institutional Review Boards of the Johns Hopkins Bloomberg School of Public Health, USA; International Centre for Diarrheal Disease Research, Bangladesh and the Centre for Injury Prevention Research, Bangladesh (ethical approval code: 00004746).

## 3. Results

Overall, 21.6% of the respondents were less than 10 years of age, 72.6% were 10 to 65 years of age and only 5.9% were more than 65 years old (Table 1) [27]. Around 60% of the respondents had received at least primary or secondary education. Around 78% were unemployed of which 27% were students; employed individuals were involved in agricultural activities (9%), skilled work (7.7%) and business (5.3%). The contribution of respondents by sub-district was Matlab North (22.8%), Matlab South (18.2%), Chandpur Sadar (11.0%) of Chandpur district, Sherpur Sadar (19.4%) of Sherpur district, Manohardi (17.3%) of Narshingdi district and Raiganj (8.8%) of Sirajgonj district.

A total of 1,159,966 individuals were included in the study of which 8.7% had sustained at least one injury in the six months preceding the date of the interview (Table 2). The total number of non-fatal injury events recorded were 115,385, of which 6.5% (*n* = 76,469) were in the low severity tertile; 2.1% (*n* = 24,018) in medium and 1.3% (*n* = 14,898) were included in the high injury severity tertile.

First aid from any provider was received for 81.7% (*n* = 94,232) of all recorded non-fatal injury events and was slightly more frequent low (82.5%) or medium (81.5%) severity injuries compared to injuries that were very severe (77.9%) (*p* value < 0.001) (Table 3). The proportion of people receiving first aid from medically trained providers increased as the severity of the injury increased (*p* value < 0.001). About 7.1% of those who received first aid from a medically trained provider were in the high severity category, as compared to only 1.5% in the low severity category. The situation was reverse for those receiving first aid from a non-medically trained provider −81.4% of those with low severity injury sought care from a non-medical provider compared to 72.9% of high severity injuries. The difference in obtaining first aid from both kinds of provider by severity category was found to be significant (Table 3).

Among all injury severity categories, receiving first aid was more common for fall injuries (39%), followed by cuts (23.4%) and injuries sustained from a blunt object (9.4%). Among those who received first aid, just over half (52%) were aged 25–64 years, and 59.3% residents were from Chandpur and Comilla districts (Table 4).

The hospitalized non-fatal injured persons of high severity who received first aid were either improving (62.6%) or had recovered (33.2%). The largest proportion of patients for all the outcomes were 25 to 64 years of age and were male. Among the 108 severe non-fatal injury patients that reported no improvement, only 8.3% saw a medically trained provider, while about two-thirds (65.7%) received first aid treatment from an untrained provider. Of those cases with no improvement, almost two-third was reported to have sustained injuries due to falls and road traffic incidents. Among the 1582 patients that were reportedly improving, 930 (58.8%) went to untrained provider for treatment and only 234 (14.8%) received first aid treatment from medically trained providers. Falls (29.7%), transport injury (27.4%) and violence (21.4%) were the commonest mechanisms of injury reported among this group. Of the 838 participants who recovered, only 160 (19.1%) received treatment from medically trained provider and 479 (57.2%) went to untrained provider for first-aid treatment. The most common causes of severe non-fatal injury among those who recovered were transport injuries (24.2%), falls (24.1%) and violence (23.2%) (Table 5).

Those non-fatal injury cases that had received first aid from a medically trained provider were more likely to recover or were in the process of improvement compared to those who received first aid from an untrained provider (OR 1.28; 95% CI 1.02–1.61) (Table 6). The chances of recovery were significantly higher among patients in Sherpur (OR 2.05; 95% CI 1.62–2.60) and Narshingdi (OR 1.98; 95% CI 1.55–2.54) districts, as compared to Chandpur/Comilla districts. However, the odds of recovery were less among those who received surgical intervention (OR 0.55; 95% CI 0.45–0.68), participants aged 25 years of age and older compared to children 10 years of age or less (OR 0.55; 95% CI 0.33–0.92) and among retired person/housewives compared to skilled laborers (OR 0.71; 95% CI 0.51–0.99).

Treatment outcome was not significantly different for those who received first aid from a non medically trained provider, as compared with those who did not receive first aid (Table 7).

## 4. Discussion

Our study is one of the largest cross-sectional census in a developing country, covering more than 1 million people from different geographical areas in Bangladesh. About 8.7% of the surveyed population had at least one injury in the six months preceding the date of the interview. Overall, 81.7% of injury events received first aid from any provider, 79% of whom were not medically trained and 2.6% medically trained. Those who received first aid from a medically trained provider irrespective of age, sex, surgical intervention, occupation, SES, geographical location and education were 1.3 times more likely to recover or be in the process of improvement compared to those who did not receive first aid from trained providers.

We found that receiving first aid is quite common in rural areas of Bangladesh with over four-fifth of the events receiving first aid increasing as severity of injury increased. Our results suggest that first aid may be beneficial, and may reduce the severity of injuries, recovery time and improve survival. There may be other factors that influences the association of first aid and outcomes such as family support, transportation, cost of treatment and responses of health facility. Our results suggest first aid may play a role to reduce the severity of injuries and improves chances of survival [14]. Our study showed that first aid treatment from trained providers increased chances of recovery among severely injured individuals and hospitalized patients implying the importance of appropriate or correct first aid. Correct first aid treatment has been reported to reduce mortality by 1.8–4.5% for trauma events [23].

We found worse outcomes for patients who were housewives, as compared to skilled labor. This may be due to housewives being at greater risk of burn injuries, which are associated with worse prognosis. This underlines that outcome depends on type of injury [28,29]. Worse treatment outcomes were also found for those who obtained surgical treatment. This may be due to delays in obtaining surgery, already poor prognosis or postoperative surgical complications. We also found worse outcomes for older age groups. This may be due to falls which are common and devastating problems associated with identifiable risk factors like weakness, unsteady gait, confusion and medications [30]. Age has been identified as one of the most significant factors in determining outcomes after traumatic injuries and head injuries [31,32,33].

We found that the proportion who received first aid increased as injury severity increased. The more severe an injury was, the more people sought first aid from a medically trained provider. A review conducted on the recognition of childhood illness and care seeking behaviour in developing countries identified six studies all of which reported that if the caregiver perceived the child’s illness as severe then they were more likely to seek care from trained providers [34]. Similar associations were found when severity of illness was defined by clinical criteria, such as rapid breathing, chest in-drawing [34].

In this study, largely untrained lay people present at the site of the event were the most common primary contacts that provided first aid to the injured. In addition, village doctors in rural Bangladesh are most commonly sought for medical care despite the existence of trained community based government and non-government health workers [35]. Multi-country evaluations of Integrated Management of Childhood Illness study showed that despite providing training for community based village health workers and availability of drugs in first level government facilities, care seeking for children under five years of age remained high from village doctors, with more than four-fifth receiving first aid treatment from an untrained provider [36]. Despite potential lack of relevant training of village doctors, when seeking medical care, the persistent use of them underlie an important consideration, when planning any intervention that seeks to improve first aid capacity through the availability of alternative trained health workers. These cultural preferences may negatively influence the acceptability and uptake of community-based first aid providers. Such potential intervention should consider cultural preference of service providers in order to maximize acceptability and uptake of interventions. Knowledge of first aid among lay people is often very limited and leads to harmful practices. A study to assess the knowledge of mothers on first aid for injuries to children arising from home accidents revealed that mothers answered an average of 11.0 (SD 5.3) out of 29 questions on first aid correctly [37]. In our study treatment outcome was not significantly different for those who received first aid from a non-medically trained provider, as compared with those who did not receive first aid and also demonstrated that those who received first aid from an untrained provider needed more time to recover reaffirming the importance of appropriateness of the first aid provided. Poor knowledge on appropriate first aid was also evident in studies conducted in developed countries [38]. A systematic review of first aid provided by lay people on trauma victims found that incorrect first aid was provided to 83.7% of cases [23]. Similarly, descriptive studies for common unintentional injuries such as burns, cuts, falls, suffocation among children in Turkey, South Africa, Ghana and Saudi-Arabia revealed that majority of the subjects had been treated with inappropriate interventions such as kitchen ingredients (yogurt, raw egg whites, honey, tomato paste) and household materials (toothpaste, aloe vera, Lavender oil) [39,40,41,42]. Although our study did not assess the appropriateness of the first aid, the negative findings associated with untrained providers raises questions on whether they provided appropriate first aid or not. A prior study from Bangladesh suggested that untrained providers mostly depend on secret spells and other ‘spiritual’ approaches with no physiological basis when providing first aid, and the custom is prevalent in both urban and rural areas [43].

Several studies indicate that training laypersons is beneficial and governments must have a more dynamic approach by promoting compulsory first aid education for example, in schools, when applying for a driving license, in the workplace and community with appropriate refresher courses [23,44,45,46,47,48,49]. In Bangladesh, CIPRB is implementing a community based first responder program in northern districts of Bangladesh [50]. The influence of SES on outcomes must also be considered during the implementation of community-based program. However, there is also need for rigorous studies to inform policy makers on the effectiveness of training on first aid.

In this census we found that the chances of recovery were significantly higher among patients in Sherpur districts compared to Chandpur/Comilla districts. This may be due availability of long-standing community-based injury intervention program, such as the first responder program by CIPRB, which created awareness [50].

### Limitations

This study did not collect data on what procedures were applied as first aid and whether the procedures that were implemented as part of first aid provided were appropriate or not. Additionally, age, education, socioeconomic status, having paid employment, source of knowledge about first aid and having attended a training course on first aid have been reported as significant predictors of knowledge and practice among first aid providers [37,38,39]. However, such information was not collected within the scope of this study and provides potential for future research in establishing an association between first aid providers and injury outcomes.

## 5. Conclusions

This large population-based survey on injury in developing countries demonstrated that for non-fatal injury, untrained providers primarily provide first aid and surgical care. Public health interventions should be designed to develop the skills of the untrained providers as well as medically trained providers. Further, policies are needed to increase access to medically trained providers for the provision of first-aid for injuries. Such intervention needs to take into account cultural preferences for service providers in order to maximize acceptability and uptake of treatments.

## Figures and Tables

**Table 1:** Socio-demographic characteristics of the rural population from seven sub-districts of Bangladesh, 2013.

Characteristics	*n* = 1,159,966	%
Age (in years)		
<10 years	250,173	21.6
10–14 years	141,725	12.2
15–17 years	61,939	5.3
18–24 years	133,161	11.5
25–64 years	504,850	43.5
≥65 years	68,118	5.9
Sex		
Male	562,721	48.5
Female	597,245	51.5
Education (*n* = 1,159,815)		
No education	291,021	25.1
Primary complete (5 years)	405,633	35.0
Secondary complete (10 years)	288,465	24.9
Secondary+	63,595	5.5
Under 5 children	111,101	9.6
Occupation (*n* = 1,159,230)		
Skilled labor (Professional)	88,645	7.6
Agriculture	103,387	8.9
Business	61,166	5.3
Unskilled/domestic (Unskilled)	24,327	2.1
Rickshaw/bus (Transport worker)	16,921	1.5
Students	311,587	26.9
Retired/unemployed/housewife	404,765	34.9
Not applicable (children & others)	148,432	12.8
Marital Status		
Married (Reference)	566,268	48.8
Never Married	226,666	19.5
Widowed/Divorced/Separated	57,390	4.9
Not applicable	309,642	26.7
Wealth quintile		
Lowest	209,500	18.1
Low	216,906	18.7
Middle	236,547	20.4
High	245,820	21.2
Highest	251,191	21.6
Sub-district		
Matlab North	264,315	22.8
Matlab South	208,443	18.0
Chandpur Sadar	127,743	11.0
Raiganj	102,526	8.8
Sherpur Sadar	226,677	20.0
Manohardi	202,092	17.4
Daudkandi	28,170	2.4
District		
Chandpur/comilla	628,671	54.2
Sirajganj	102,526	8.8
Sherpur	226,677	19.5
Narshingdi	202,092	17.4

**Table 2:** Number of individuals and non-fatal injury events by injury severity, 2013.

Characteristics	Number of Injury Events (*n* = 1,173,974)		Number of Individuals (*n* = 1,159,966)	
	*n*	%	*n*	%
No Injury	1,058,589	90.17	1,058,589	91.26
Non-Fatal Injury severity	115,385	9.83	101,377	8.74
Low	76,469	6.51	66,430	5.73
Medium	24,018	2.05	21,453	1.85
High	14,898	1.27	13,494	1.16

**Table 3:** Percentage of non-fatal injury events that received first-aid according to injury severity categories, 2013.

Severity Category	Received First Aid *n* (%) ^1^		Received First Aid from Medically Trained Provider *n* (%) ^1^		Received First Aid from Non-Medically Trained Providers *n* (%) ^1^	
	Yes	No	Yes	No	Yes	No
Low	63,058 (82.5)	13,411 (17.5)	1175 (1.5)	75,294 (98.5)	62,282 (81.4)	14,187 (18.6)
Medium	19,572 (81.5)	4446 (18.5)	756 (3.1)	23,262 (96.9)	19,049 (79.3)	4969 (20.7)
High	11,602 (77.9)	3296 (22.1)	1053 (7.1)	13,845 (92.9)	10,858 (72.9)	4040 (27.1)
Total	94,232 (81.7)	21,153 (18.3)	2984 (2.6)	112,401 (97.4)	92,189 (79.9)	23,196 (20.1)

^1^ All significant at *p*-value of <0.001.

**Table 4:** The percentage of non-fatal injury events that received first aid by type of provider, injury mechanism, socio-demographic and geographical factors among different non-fatal injury severity categories.

Characteristics	Injury Severity Tertiles			
	Low *n* (%)	Medium *n* (%)	High *n* (%)	Total *n* (%)
*n* (Number of injury events that received first aid)	63,058 (100)	19,572 (100)	11,602 (100)	94,232 (100)
External cause of injury				
Attempted suicide	18 (0.0)	2 (0.0)	19 (0.2)	39 (0.0)
Transport injury	3596 (5.7)	2079 (10.6)	1379 (11.9)	7054 (7.5)
Violence	1087 (1.7)	486 (2.5)	790 (6.8)	2363 (2.5)
Fall	22,685 (36.0)	7793 (39.8)	6288 (54.2)	36,766 (39.0)
Cut injury	16,815 (26.7)	4508 (23.0)	732 (6.3)	22,055 (23.4)
Burn	3948 (6.3)	1386 (7.1)	200 (1.7)	5534 (5.9)
Drowning	1778 (2.8)	59 (0.3)	600 (5.2)	2437 (2.6)
Unintentional poisoning	19 (0.0)	8 (0.0)	39 (0.3)	66 (0.1)
Machine injury	566 (0.9)	270 (1.4)	133 (1.2)	969 (1.0)
Electrocution	401 (0.6)	74 (0.4)	190 (1.6)	665 (0.7)
Animal bite injury	5974 (9.5)	870 (4.5)	402 (3.5)	7246 (7.7)
Injury by blunt object	6064 (9.6)	2029 (10.4)	799 (6.9)	8892 (9.4)
Suffocation	107 (0.2)	8 (0.0)	31 (0.3)	146 (0.2)
Age (in years)				
<10 years	12,865 (20.4)	3028 (15.5)	2428 (20.9)	18,321(19.4)
10–14 years	6324 (10.0)	1729 (8.8)	977 (8.4)	9030 (9.6)
15–17 years	2530 (4.0)	788 (4.0)	375 (3.2)	3693 (3.9)
18–24 years	5412 (8.6)	1624 (8.3)	787 (6.8)	7823 (8.3)
25–64 years	32,259 (51.2)	10,788 (55.1)	5936 (51.2)	48,983 (52.0)
≥65 years	3668 (5.8)	1615 (8.3)	1099 (9.5)	6382 (6.8)
Sub-district				
Matlab North	13,109 (20.8)	5592 (28.6)	3011 (26.0)	21,712 (23.0)
Matlab South	14,282 (22.7)	4356 (22.3)	2828 (24.4)	21,466 (22.8)
Chandpur Sadar	4416 (7.0)	2642 (13.5)	1487 (12.8)	8545 (9.1)
Raiganj	9507 (15.1)	1735 (8.9)	1233 (10.6)	12,475 (13.2)
Sherpur	9725 (15.4)	2026 (10.4)	1467 (12.6)	13,218 (14.0)
Manohardi	9077 (14.4)	2362 (12.1)	1211 (10.4)	12,650 (13.4)
Daud Kandi	2942 (4.7)	859 (4.4)	365 (3.2)	4166 (4.4)
District				
Chandpur/Comilla	34,749 (55.1)	13,449 (68.7)	7691 (66.3)	55,889 (59.3)
Sirajgonj	9507 (15.1)	1735 (8.9)	1233 (10.6)	12,475 (13.2)
Sherpur	9725 (15.4)	2026 (10.4)	1467 (12.6)	13,218 (14.0)
Narshingdi	9077 (14.4)	2362 (12.1)	1211 (10.4)	12,650 (13.4)

**Table 5:** The distribution of treatment outcomes by injury mechanism, socio-demographic and geographical factors among severe non-fatal hospitalized injury patients.

Characteristics	Treatment Outcome					
	No Improvement		Improving		Recovered	
	*n*	%	*n*	%	*n*	%
Severe non-fatal hospitalized injury events who received first aid	108	4.3	1582	62.6	838	33.2
Received first aid from Medically trained provider						
No	99	91.7	1348	85.2	678	80.9
Yes	9	8.3	234	14.8	160	19.1
Received first aid from non-medically trained providers						
No	37	34.3	652	41.2	359	42.8
Yes	71	65.7	930	58.8	479	57.2
External cause of severe non-fatal injury						
Attempted suicide	0	0.0	9	0.6	6	0.7
Transport injury	33	30.6	433	27.4	203	24.2
Violence	15	13.9	339	21.4	194	23.2
Fall	36	33.3	470	29.7	202	24.1
Cut injury	4	3.7	91	5.8	68	8.1
Burn	3	2.8	37	2.3	23	2.7
Drowning	0	0.0	14	0.9	17	2.0
Unintentional poisoning	0	0.0	7	0.4	7	0.8
Machine injury	2	1.9	31	2.0	18	2.1
Electrocution	2	1.9	25	1.6	21	2.5
Animal bite injury	2	1.9	38	2.4	29	3.5
Injury by blunt object	11	10.2	87	5.5	49	5.8
Suffocation	0	0.0	1	0.1	1	0.1
Age (in years)						
<10 years	6	5.6	168	10.6	113	13.5
10–14 years	5	4.6	94	5.9	53	6.3
15–17 years	7	6.5	68	4.3	39	4.7
18–24 years	9	8.3	165	10.4	96	11.5
25–64 years	66	61.1	946	59.8	485	57.9
≥65 years	15	13.9	141	8.9	52	6.2
Sex						
Male	71	65.7	1099	69.5	598	71.4
Female	37	34.3	483	30.5	240	28.6
Sub-district						
Matlab North	25	23.1	384	24.3	224	26.7
Matlab South	15	13.9	411	26.0	96	11.5
Chandpur Sadar	22	20.4	243	15.4	85	10.1
Raiganj	27	25.0	103	6.5	87	10.4
Sherpur	8	7.4	227	14.3	189	22.6
Manohardi	8	7.4	180	11.4	151	18.0
Daudkandi	3	2.8	34	2.1	6	0.7
District						
Chandpur/Comilla	65	60.2	1072	67.8	411	49.0
Sirajgonj	27	25.0	103	6.5	87	10.4
Sherpur	8	7.4	227	14.3	189	22.6
Narshingdi	8	7.4	180	11.4	151	18.0

**Table 6:** Multivariate analysis of treatment outcomes by trained provider among severe non-fatal hospitalized injuries.

Characteristics	Treatment Outcome (1 = Recovered/Improving; 0 = No Improvement)			
	Unadjusted		Adjusted	
	OR	95% CI	OR	95% CI
Received first aid from trained provider				
Yes	1.44 *	1.16–1.79	1.28 *	1.02–1.61
No	Reference group		Reference group	
Surgical intervention				
Yes	0.52 *	0.43–0.63	0.55 *	0.45–0.68
No	Reference group		Reference group	
External cause of severe non-fatal injury				
Attempted suicide	Reference group		Reference group	
Transport injury	0.6	0.22–1.64	0.613	0.22–1.72
Violence	0.78	0.28–2.14	0.902	0.32–2.52
Fall	0.55	0.20–1.52	0.652	0.23–1.83
Cut injury	1.00	0.35–2.85	0.975	0.34–2.82
Burn	0.77	0.25–2.36	0.650	0.21–2.05
Drowning	1.74	0.51–5.87	1.499	0.42–5.34
Unintentional poisoning	1.45	0.34–6.06	1.435	0.33–6.19
Machine injury	0.75	0.24–2.36	0.904	0.28–2.91
Electrocution	1.04	0.33–3.28	0.953	0.30–3.07
Animal bite injury	1.01	0.33–3.03	0.906	0.29–2.80
Injury by blunt object	0.63	0.22–1.80	0.776	0.26–2.28
Suffocation	1.45	0.08–26.26	1.406	0.08–26.17
Age (in years)				
<10 years	Reference group		Reference group	
10–14 years	0.81	0.54–1.21	0.78	0.48–1.25
15–17 years	0.73	0.47–1.14	0.68	0.39–1.19
18–24 years	0.83	0.59–1.16	0.73	0.43–1.23
25–64 years	0.71 *	0.55–0.92	0.55 **	0.33–0.92
≥65 years	0.48 *	0.33–0.69	0.45 *	0.25–0.81
Sex				
Male	Reference group		Reference group	
Female	0.89	0.74–1.06	1.13	0.89–1.45
Wealth quintile				
Lowest	Reference group		Reference group	
Low	1.00	0.77–1.29	1.06	0.81–1.39
Middle	1.07	0.83–1.38	1.15	0.88–1.50
High	0.86	0.66–1.11	0.95	0.73–1.25
Highest	0.89	0.68–1.16	1.08	0.81–1.44
District				
Chandpur/comilla	Reference group		Reference group	
Sirajganj	1.38 **	1.02–1.87	1.29	0.94–1.78
Sherpur	2.16 *	1.74–2.69	2.05 *	1.62–2.60
Narshingdi	2.14 *	1.69–2.71	1.98 *	1.55–2.54
Occupation				
Skilled labor (Professional)	Reference group		Reference group	
Agriculture	1.23	0.92–1.65	1.18	0.86–1.60
Business	0.97	0.68–1.37	0.97	0.68–1.39
Unskilled/domestic (Unskilled)	1.04	0.66–1.62	0.99	0.62–1.56
Rickshaw/bus (Transport worker)	1.32	0.87–2.01	1.30	0.84–2.01
Students	1.07	0.80–1.44	0.75	0.48–1.18
Retired/unemployed/housewife	0.71 **	0.55–0.93	0.71 **	0.51–0.99
Not applicable (children & others)	1.33	0.92–1.92	0.66	0.32–1.38
Education				
No education	Reference group		Reference group	
Primary	0.98	0.80–1.20	0.97	0.78–1.21
Secondary	0.92	0.74–1.14	0.96	0.74–1.24
A levels/college/Advanced/Professional Degree	0.85	0.58–1.25	0.95	0.61–1.48
Not applicable (U5 children)	1.36	0.94–1.98	1.09	0.51–2.30

* *p* < 0.01, ** *p* < 0.05.

**Table 7:** Multivariate analysis of treatment outcomes by un-trained provider among severe non-fatal hospitalised injuries.

Characteristics	Treatment Outcome (1 = Recovered/Improving; 0 = No Improvement)			
	Unadjusted		Adjusted	
	OR	95% CI	OR	95% CI
Receiver first aid from untrained Provider				
Yes	0.89	0.76–1.05	0.90	0.76–1.06
No	Reference group		Reference group	
Surgical intervention				
Yes	0.52 *	0.43–0.63	0.55 *	0.45–0.67
No	Reference group		Reference group	
External cause of severe non-fatal injury				
Attempted suicide/suicide	Reference group		Reference group	
Transport injury	0.60	0.22–1.64	0.611	0.21–1.71
Violence	0.78	0.28–2.14	0.896	0.31–2.51
Fall	0.55	0.20–1.52	0.657	0.23–1.84
Cut injury	1.00	0.35–2.85	0.990	0.34–2.86
Burn	0.77	0.25–2.36	0.657	0.20–2.07
Drowning	1.74	0.51–5.87	1.558	0.43–5.55
Unintentional poisoning	1.45	0.34–6.06	1.530	0.35–6.58
Machine injury	0.75	0.24–2.36	0.903	0.28–2.90
Electrocution	1.04	0.33–3.28	0.950	0.29–3.05
Animal bite injury	1.01	0.33–3.03	0.913	0.29–2.82
Injury by blunt object	0.63	0.22–1.80	0.779	0.26–2.28
Suffocation	1.45	0.08–26.26	1.373	0.07–25.63
Age (in years)				
<10 years	Reference group		Reference group	
10–14 years	0.81	0.54–1.21	0.78	0.48–1.24
15–17 years	0.73	0.47–1.14	0.69	0.39–1.19
18–24 years	0.83	0.59–1.16	0.74	0.43–1.24
25–64 years	0.71 *	0.55–0.92	0.55 **	0.33–0.92
≥65 years	0.48 *	0.33–0.69	0.46 **	0.25–0.83
Sex				
Male	Reference group		Reference group	
Female	0.89	0.74–1.06	1.117285	0.87–1.42
Wealth quintile				
Lowest	Reference group		Reference group	
Low	1.00	0.77–1.29	1.06	0.80–1.38
Middle	1.07	0.83–1.38	1.15	0.88–1.50
High	0.86	0.66–1.11	0.95	0.72–1.24
Highest	0.89	0.68–1.16	1.10	0.82–1.46
District				
Chandpur/comilla	Reference group		Reference group	
Sirajganj	1.38 **	1.02–1.87	1.33	0.97–1.84
Sherpur	2.16 *	1.74–2.69	2.14	1.69–2.69
Narshingdi	2.14 *	1.69–2.71	2.03	1.58–2.59
Occupation				
Skilled labor (Professional)	Reference group		Reference group	
Agriculture	1.23	0.92–1.65	1.17	0.85–1.59
Business	0.97	0.68–1.37	0.96	0.67–1.37
Unskilled/domestic (Unskilled)	1.04	0.66–1.62	0.99	0.62–1.56
Rickshaw/bus (Transport worker)	1.32	0.87–2.01	1.29	0.83–1.99
Students	1.07	0.80–1.44	0.77	0.49–1.19
Retired/unemployed/housewife	0.71 **	0.55–0.93	0.72	0.52–1.00
Not applicable (children & others)	1.33	0.92–1.92	0.69	0.33–1.42
Education				
No education	Reference group		Reference group	
Primary	0.98	0.80–1.20	0.97	0.77–1.21
Secondary	0.92	0.74–1.14	0.96	0.73–1.23
A levels/college/Advanced/Professional Degree	0.85	0.58–1.25	0.94	0.60–1.45
Not applicable (U5 children)	1.36	0.94–1.98	1.05	0.49–2.23

* *p* < 0.01, ** *p* < 0.05.

## Abbreviations

The following abbreviations are used in this manuscript:

LMICs	Low- and middle-income countries
OR	Odds ratio
CI	Confidence Interval
SOLID	Saving of children’s Lives from Drowning
BHIS	Bangladesh Health and Injury Survey
IFRC	International Federation of Red Cross and Red Crescent Societies
SOLID	Saving of children’s lives from drowning
CIPRB	Center for Injury Prevention and Research, Bangladesh

## References

[1] Gosselin R.A., Spiegel D.A., Coughlin R., Zirkle L.G. (2009). Injuries: The neglected burden in developing countries. Bull. World Health Org..

[2] Jha P., Chaloupka F.J., Moore J., Gajalakshmi V., Gupta P.C., Peck R., Jamison D., Breman J., Measham A., Alleyne G. Disease Control Priorities in Developing Countries 2006. http://citeseerx.ist.psu.edu/viewdoc/download?doi=10.1.1.668.8031&rep=rep1&type=pdf.

[3] Stewart K.-A.A., Groen R.S., Kamara T.B., Farahzad M.M., Samai M., Cassidy L.D., Kushner A.L., Wren S.M. (2013). Traumatic injuries in developing countries: Report from a nationwide cross-sectional survey of Sierra Leone. JAMA Surg..

[4] Murray C.J., Vos T., Lozano R., Naghavi M., Flaxman A.D., Michaud C., Ezzati M., Shibuya K., Salomon J.A., Abdalla S. (2013). Disability-adjusted life years (DALYs) for 291 diseases and injuries in 21 regions, 1990–2010: A systematic analysis for the Global Burden of Disease Study 2010. Lancet.

[5] Wang H.D., Naghavi M., Allen C., Barber R.M., Bhutta Z.A., Carter A., Casey D.C., Charlson F.J., Chen A.Z., Coates M.M. (2016). Global, regional, and national life expectancy, all-cause mortality, and cause-specific mortality for 249 causes of death, 1980–2015: A systematic analysis for the Global Burden of Disease Study 2015. Lancet.

[6] Vos T., Allen C., Arora M., Barber R.M., Bhutta Z.A., Brown A., Carter A., Casey D.C., Charlson F.J., Chen A.Z. (2016). Global, regional, and national incidence, prevalence, and years lived with disability for 310 diseases and injuries, 1990–2015: A systematic analysis for the Global Burden of Disease Study 2015. Lancet.

[7] Mahajan N., Aggarwal M., Raina S., Verma L.R., Mazta S.R., Gupta B. (2013). Pattern of non-fatal injuries in road traffic crashes in a hilly area: A study from Shimla, North India. Int. J. Crit. Illness Injury Sci..

[8] Peltzer K., Phaswana-Mafuya N., Arokiasamy P., Biritwum R., Yawson A., Minicuci N., Williams J.S., Kowal P., Chatterji S. (2015). Prevalence, circumstances and consequences of non-fatal road traffic injuries and other bodily injuries among older people in China, Ghana, India, Mexico, Russia and South Africa. Afr. Saf. Promot..

[9] Molcho M., Harel Y., Pickett W., Scheidt P.C., Mazur J., Overpeck M.D., HBSC Violence and Injury Writing Group (2006). The epidemiology of non-fatal injuries among 11-, 13- and 15-year old youth in 11 countries: Findings from the 1998 WHO-HBSC cross national survey. Int. J. Injury Control Saf. Promot..

[10] Lescohier I., Gallagher S.S. (1996). Unintentional injury. Handbook of Adolescent Health Risk Behavior.

[11] Rivara F.P., Grossman D.C., Cummings P. (1997). Injury prevention. N. Engl. J. Med..

[12] Odero W., Garner P., Zwi A. (1997). Road traffic injuries in developing countries: A comprehensive review of epidemiological studies. Trop. Med. Int. Health.

[13] Birgul P., Ocaktan M.E., Akdur R., Soner Y.M., Sevil I., Safa C. (2013). Evaluation of unintentional injuries sustained by children: A hospital based study from Ankara-Turkey. Pak. J. Med. Sci..

[14] IFRC First Aid for a Safer Future: Updated Global Edition—Advocacy Report 2010. http://www.ifrc.org/PageFiles/53459/First%20aid%20for%20a%20safer%20future%20Updated%20global%20edition%20%20Advocacy%20report%202010%20(2).pdf?epslanguage=en.

[15] Rahman A., Shafinaz S., Linnan M. (2005). Bangladesh Health and Injury Survey-Report on Children.

[16] Giashuddin S.M., Rahman A., Rahman F., Mashreky S.R., Chowdhury S.M., Linnan M., Shafinaz S. (2009). Socioeconomic inequality in child injury in Bangladesh—Implication for developing countries. Int. J. Equity Health.

[17] Wood F.M., Phillips M., Jovic T., Cassidy J.T., Cameron P., Edgar D.W. (2016). Water first aid is beneficial in humans post-burn: Evidence from a bi-national cohort study. PLoS ONE.

[18] Larry L.G., Scheulen J.J., Munster A.M. (1982). Chemical burns: Effect of prompt first aid. J. Trauma Acute Care Surg..

[19] IFRC (2015). Law and First Aid: Promoting and Protecting Life-Saving Action.

[20] Shotland R.L., Heinold W.D. (1985). Bystander response to arterial bleeding: Helping skills, the decision-making process, and differentiating the helping response. J. Personal. Soc. Psychol..

[21] Wei Y.-L., Chen L.-L., Li T.-C., Ma W.-F., Peng N.-H., Huang L.-C. (2013). Self-efficacy of first aid for home accidents among parents with 0-to 4-year-old children at a metropolitan community health center in Taiwan. Accid. Anal. Prev..

[22] Arbon P., Hayes J., Woodman R. (2011). First aid and harm minimization for victims of road trauma: A population study. Prehosp. Disaster Med..

[23] Tannvik T., Bakke H., Wisborg T. (2012). A systematic literature review on first aid provided by laypeople to trauma victims. Acta Anaesthesiol. Scand..

[24] Sahu S.A., Agrawal K., Patel P.K. (2016). Scald burn, a preventable injury: Analysis of 4306 patients from a major tertiary care center. Burns.

[25] Hyder A.A., Alonge O., He S., Wadhwaniya S., Rahman F., Rahman A., Arifeen S.E. (2014). Saving of Children’s Lives from Drowning Project in Bangladesh. Am. J. Prev. Med..

[26] Hyder A.A., Alonge O., He S., Wadhwaniya S., Rahman F., Rahman A., Arifeen S.E. (2014). A Framework for Addressing Implementation Gap in Global Drowning Prevention Interventions: Experiences from Bangladesh. J. Health Popul. Nutr..

[27] Alonge O., Agrawal P., Talab A., Rahman Q.A., Rahman F., Arifeen S.E., Hyder A.A. (2017). Fatal and Non-Fatal Injury Outcomes: Results from a Purposively Sampled Census of Seven Rural Sub-Districts in Bangladesh. Lancet Glob. Health.

[28] Edelman L.S. (2007). Social and economic factors associated with the risk of burn injury. Burns.

[29] Forjuoh S.N. (2006). Burns in low-and middle-income countries: A review of available literature on descriptive epidemiology, risk factors, treatment, and prevention. Burns.

[30] Rubenstein L.Z. (2006). Falls in older people: Epidemiology, risk factors and strategies for prevention. Age Ageing.

[31] Demetriades D., Murray J., Martin M., Velmahos G., Salim A., Alo K., Rhee P. (2004). Pedestrians injured by automobiles: Relationship of age to injury type and severity. J. Am. Coll. Surg..

[32] Luerssen T.G., Klauber M.R., Marshall L.F. (1988). Outcome from head injury related to patient’age: A longitudinal prospective study of adult and pediatric head injury. J. Neurosurg..

[33] Disease Control Priorities Project (2006). Disease Control Priorities in Developing Countries.

[34] Geldsetzer P., Williams T.C., Kirolos A., Mitchell S., Ratcliffe L.A., Kohli-Lynch M.K., Bischoff E.J.L., Cameron S., Campbell H. (2014). The recognition of and care seeking behaviour for childhood illness in developing countries: A systematic review. PLoS ONE.

[35] Mahmood S.S., Iqbal M., Hanifi S., Wahed T., Bhuiya A. (2010). Are “Village Doctors” in Bangladesh a curse or a blessing?. BMC Int. Health Hum. Rights.

[36] El Arifeen S., Blum L.S., Hoque D.M., Chowdhury E.K., Khan R., Black R.E., Victora C.G., Bryce J. (2004). Integrated Management of Childhood Illness (IMCI) in Bangladesh: Early findings from a cluster-randomised study. Lancet.

[37] Eldosoky R.S. (2012). Home-related injuries among children: Knowledge, attitudes and practice about first aid among rural mothers. East. Mediterr. Health J..

[38] Davies M., Maguire S., Okolie C., Watkins W., Kemp A.M. (2013). How much do parents know about first aid for burns?. Burns.

[39] Alomar M., Rouqi F.A., Eldali A. (2016). Knowledge, attitude, and belief regarding burn first aid among caregivers attending pediatric emergency medicine departments. Burns.

[40] Karaoz B. (2010). First-aid home treatment of burns among children and some implications at Milas, Turkey. J. Emerg. Nurs..

[41] Jonkheijm A., Zuidgeest J.J., van Dijk M., van As A.B. (2013). Childhood unintentional injuries: Supervision and first aid provided. Afr. J. Paediatr. Surg..

[42] Gyedu A., Mock C., Nakua E., Otupiri E., Donkor P., Ebel B.E. (2015). Pediatric First Aid Practices in Ghana: A Population-Based Survey. World J. Surg..

[43] Rahman A. Bangladesh Health and Injury Survey: Report on Children. http://www.unicef.org/bangladesh/Bangladesh_Health_and_Injury_Survey-Report_on_Children.pdf.

[44] Walsh K., Hili S., Dheansa B. (2016). Compulsory teaching of first aid in UK schools—A missed opportunity?. Burns.

[45] Reveruzzi B., Buckley L., Sheehan M. (2016). School-Based First Aid Training Programs: A Systematic Review. J. Sch. Health.

[46] Olumide A.O., Asuzu M.C., Kale O.O. (2015). Effect of First Aid Education on First Aid Knowledge and Skills of Commercial Drivers in South West Nigeria. Prehosp. Disaster Med..

[47] VanderBurgh D., Jamieson R., Beardy J., Ritchie S.D., Orkin A. (2014). Community-based first aid: A program report on the intersection of community-based participatory research and first aid education in a remote Canadian Aboriginal community. Rural Remote Health.

[48] Vakili M.A., Mohjervatan A., Heydari S.T., Akbarzadeh A., Hosini N.S., Alizad F., Arasteh P., Moghasemi M.J. (2014). The efficacy of a first aid training course for drivers: An experience from northern Iran. Chin. J. Traumatol..

[49] Oliver E., Cooper J., McKinney D. (2014). Can first aid training encourage individuals’ propensity to act in an emergency situation? A pilot study. Emerg. Med. J..

[50] Rahman A., Mecrow T.S., Mashreky S.R., Rahman A.K., Nusrat N., Khanam M., Scarr J., Linnan M. (2014). Feasibility of a first responder programme in rural Bangladesh. Resuscitation.

